# Guiding student use of generative AI in undergraduate pharmacology: a narrative review and proposed source-checking process framework

**DOI:** 10.3389/fmed.2026.1910543

**Published:** 2026-07-28

**Authors:** Xiaohang Che, Yueyang Liu

**Affiliations:** 1 Department of Pharmacology, Shenyang Pharmaceutical University, Shenyang, China; 2 Science and Experimental Research Center of Department of Laboratory Medicine, Shenyang Medical College, Shenyang, China

**Keywords:** drug information, formative assessment, generative artificial intelligence, pharmacological reasoning, pharmacology education

## Abstract

Generative artificial intelligence (AI) has entered undergraduate pharmacology learning through chatbots, writing assistants and large language model (LLM) interfaces. Students now use these tools to clarify mechanisms, compare drug classes, draft case answers, prepare questions and search for medication-related information. The same convenience creates a course-level problem: a response that is fluent and well organized may still contain an incorrect mechanism, an unsupported drug claim, a missing contraindication or a recommendation that does not fit the patient described in a case. This narrative review examines recent work on AI in medical and pharmacy education, with particular attention to pharmacology-relevant teaching tasks. It identifies problems that are especially important for pharmacology courses: hidden AI use, weak source checking of AI-generated drug claims, mechanism explanations that remain descriptive rather than causal, medication suggestions that ignore patient variables and assessment practices that evaluate the submitted answer rather than the reasoning behind it. In response, the article proposes a source-checking process for formative pharmacology coursework tasks in which AI use is permitted, including pre-class preparation, classroom case work, chapter-level tasks and short revision notes. The process asks students to define the task, check drug claims against defined sources, rebuild the pharmacological mechanism, test the answer against patient context and revise with feedback and justification. By embedding AI use within source checking, mechanism reconstruction and patient-context appraisal, this approach may help pharmacology teachers turn generated answers into structured material for learning, discussion and formative assessment.

## 1 Introduction

Undergraduate pharmacology sits between basic biomedical science and the safe use of medicines. A student must learn receptor actions, enzyme inhibition, transporters, pharmacokinetic parameters and pharmacodynamic relationships, but those facts have limited educational value if they cannot be used to explain benefits, toxicity, contraindications or monitoring. This has been a persistent difficulty in pharmacology teaching. The knowledge base is large, drug information changes quickly and students often move from memorizing drug classes to using them in cases without enough time to develop a stable reasoning process (1–3).

Generative artificial intelligence (AI) has made this problem more visible. In routine study, students can ask ChatGPT, Gemini, Claude, Copilot or another large language model (LLM) interface to summarize a chapter, list adverse effects or draft an answer to a case. Reviews and systematic reviews in pharmacy, medical and health-professions education describe similar uses for teaching-material preparation, assessment design, personalized learning, feedback and learning analytics (4–11). A prospective survey of 415 United States medical students found that 52% had used ChatGPT for medical-school coursework. Frequently reported uses included explaining medical concepts, assisting with diagnosis or treatment planning and proofreading academic work (12). The issue is therefore no longer whether students will encounter these tools. They already do. The more practical question is how pharmacology courses should respond when AI use becomes part of ordinary learning.

The response cannot be limited to a general statement about academic integrity. Pharmacology has safety-sensitive content. Drug information depends on dose, route, age, pregnancy status, organ function, allergy history, comorbidities and concomitant medicines. A general answer may help a student begin, but it can also obscure an important exception. Studies using real or simulated medication questions have reported incomplete or incorrect AI-generated answers, unsupported references, unstable responses and weak performance in patient cases, raising concerns about potential harm if such outputs are transferred uncritically into clinical or medication-related decision making (13–17). Fabricated references in AI-assisted medical writing further illustrate why source checking cannot be treated as a minor editing step (18).

Current literature has made important progress in describing AI opportunities, risks and literacy needs in health-professions education (19–21). Existing pharmacy-education studies have also examined student and faculty perceptions, AI performance on pharmacy tasks, case generation and activities designed to improve AI readiness (22–27). These studies establish that AI use is already relevant to pharmacy and pharmacology teaching. They leave a more operational question open: when AI output enters pre-class preparation, case discussion, chapter work or other formative pharmacology coursework, what exactly should the student do with that output before presenting an answer?

This narrative review first summarizes how AI is being used in pharmacology-relevant education, including mechanism explanation, case and question generation, tutoring and feedback, drug-information queries, and issues related to AI literacy and academic integrity. Across these uses, the review identifies a recurring instructional problem: students may receive fluent AI-generated answers, but the learning process behind those answers often remains unobserved. More specifically, AI use may be hidden, drug claims may go unchecked, mechanism explanations may remain descriptive, medication suggestions may be detached from patient variables, and assessment may focus on the final answer rather than the reasoning process. These problems are especially important in pharmacology because drug information is conditional, mechanism-based and safety-sensitive. On this basis, the article proposes a source-checking process for AI-permitted formative pharmacology coursework. The process asks students to record AI use, verify key drug claims, rebuild the pharmacological mechanism, test answers against patient context and revise with feedback and justification.

## 2 Methods

### 2.1 Literature selection

This work used a narrative review approach combined with conceptual framework development. PubMed served as the main biomedical database and was supplemented by Web of Science. Searches covered publications available up to July 2026. Search terms included *generative artificial intelligence, large language models, ChatGPT, Gemini, Claude, Copilot, pharmacy education, pharmacology education, health profession education*.

Titles and abstracts were screened for relevance, followed by full-text review. Publications were eligible when they examined generative AI or large language models in medical, pharmacy, pharmacology or health-professions education, or provided evidence directly relevant to undergraduate pharmacology teaching. Relevant topics included mechanism explanation, case and question generation, tutoring and feedback, drug-information activities, AI literacy, academic integrity, clinical or pharmacological reasoning, and the accuracy or reliability of AI-generated medication-related content. Publications were excluded when they focused on technical model development without an educational or medication-information application, non-health disciplines without transferable content, or clinical AI applications unrelated to teaching, learning or drug information. The searches retrieved 139 records. After title and abstract screening, 82 records were excluded. Full texts were sought for the remaining 57 records; eight were excluded because they were published in languages other than English or because the full text could not be obtained. The remaining 49 publications were reviewed in full and included in the final narrative synthesis. Clinical guidelines, regulatory documents and drug- or disease-specific evidence used only for the worked example were not included in the narrative synthesis. Foundational literature on pharmacology education, cognitive load, formative feedback, drug-information education and clinical reasoning was cited for contextual or framework-supporting purposes but was not counted as included review literature.

### 2.2 Framework development

**Figure 1:**
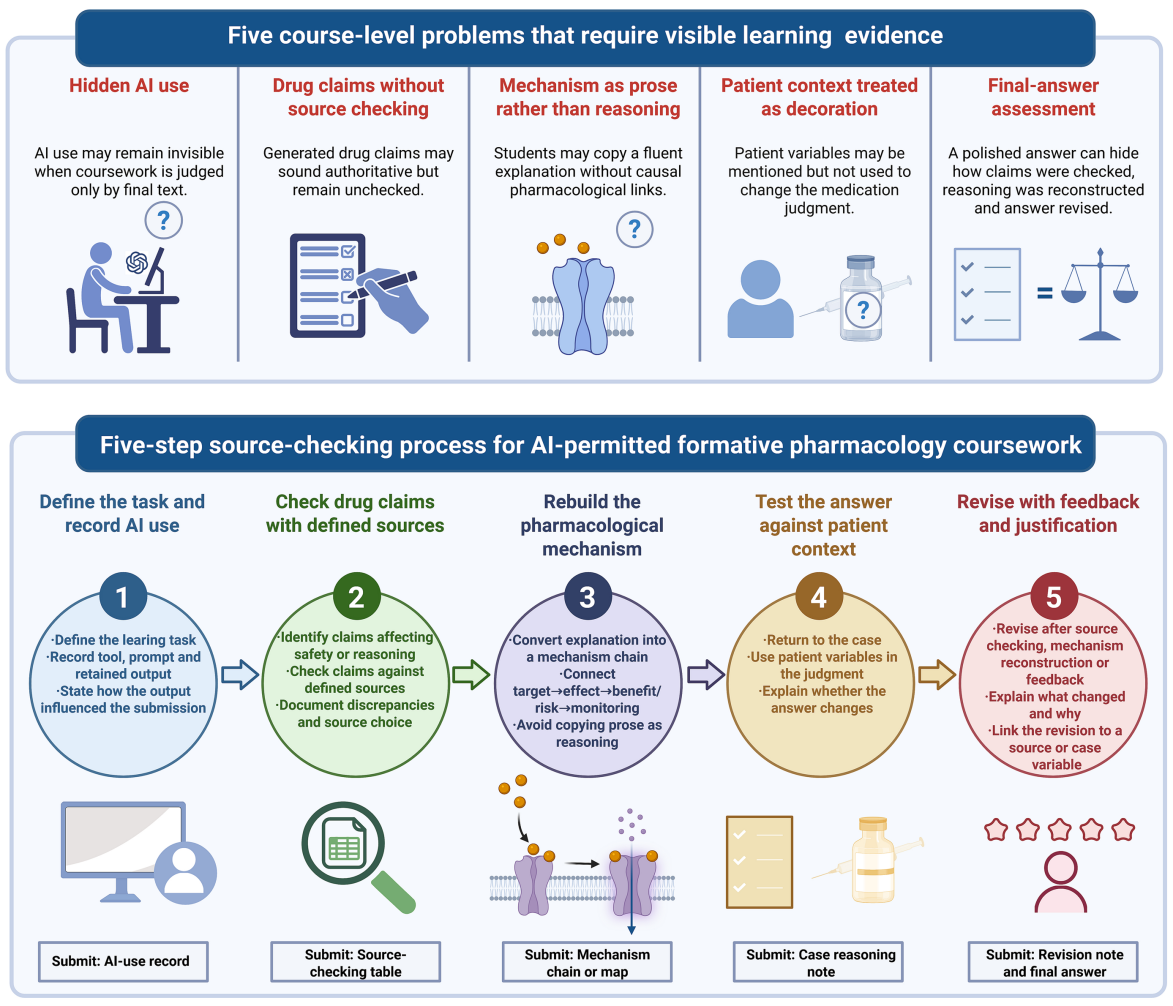
Course-level problems and source-checking process framework for AI-permitted formative undergraduate pharmacology coursework. The figure was developed by mapping five course-level problems to five student actions and corresponding forms of visible learning evidence. The figure was created with the help of BioRender (Che, X. (2026) https://BioRender.com/8prwcxb).

The synthesis proceeded in two linked stages. First, findings reported in the included literature were organized by educational use and type of limitation. These findings included inaccurate or incomplete medication-related answers, fabricated or unsupported references, variation in performance across models and prompts, and student or faculty perceptions of AI usefulness and reliability. Second, the educational implications of these findings for undergraduate pharmacology were examined. Recurrent concerns were organized into five course-level problems, and each problem was linked to a corresponding student action and an observable form of coursework evidence. This mapping produced the five-step process presented in this article and informed the parallel problem-action-evidence structure of Table 1 and Figure 1. The process draws on the narrative synthesis and established principles of pharmacological reasoning and formative assessment rather than on a single existing educational framework.

**Table 1:** Problems requiring a source-checking process in formative pharmacology coursework.

Course problem	What the student must show	Assessment evidence
Hidden AI use	Purpose of AI use, prompt and retained material.	Tool name, prompt and short AI-use record.
Drug claims without source checking	Which claims were checked and against what source.	Source-checking table.
Mechanism as prose rather than reasoning	Causal links from targets to benefits, risks and monitoring.	Mechanism chain or map.
Patient context treated as decoration	How patient variables changed the answer.	Case reasoning note.
Final-answer assessment	How the answer was corrected.	Revision note and final answer.

## 3 How AI is being used in pharmacology-relevant education

The literature suggests five common entry points for AI in pharmacology-relevant education (Table 2).

**Table 2:** Main uses and pharmacology-specific risks of AI in pharmacology-relevant education.

Use of AI	Typical student or teacher use	Main pharmacology risk	Course response
Mechanism explanation	Students ask for explanations of receptors, enzymes, pharmacokinetics or adverse reactions.	Clear wording may hide an incorrect or incomplete causal chain.	Require a checked mechanism chain rather than a copied explanation.
Case and question generation	Teachers or students generate vignettes, MCQs, comparisons or case variants.	Cases may omit variables that determine medication safety.	Teacher review for accuracy, missing patient variables and learning objective.
Tutoring and feedback	AI gives hints, quizzes or comments on answers.	Students may accept full answers and skip retrieval or revision.	Use tiered hints and require correction notes.
Drug-information queries	Students ask about indications, contraindications, interactions, monitoring or dose adjustment.	Incorrect claims, fabricated references or missing safety exceptions.	Check key drug claims against defined sources.
AI literacy and academic integrity	Students learn disclosure, privacy, prompting and recognition of AI limitations.	General AI literacy may not address medication safety.	Make drug names, interactions, special populations and monitoring explicit checking targets.

### 3.1 Mechanism explanation

One common student use of AI is to request explanations of medical concepts (28). In a prospective survey of medical students, explanation of medical concepts was among the most commonly reported educational uses of ChatGPT (12). This is understandable in pharmacology because mechanism vocabulary is dense and often abstract. An LLM can restate receptor pharmacology in simpler language, build a comparison table or offer a short example. AI-generated learning materials, including podcasts and other study resources, have also been explored in undergraduate medical pharmacology (29, 30). Such support can lower the entry barrier for students who are lost at the level of terminology.

The limitation is that a clear explanation is not always a correct one. Students may receive a mechanism that confuses target selectivity, omits pharmacokinetic issues or presents a causal chain as if all steps were equally established. Cognitive load theory is useful here: scaffolding can support learning, but unreliable scaffolding can also create a misleading schema (31, 32). Pharmacology teaching should therefore ask students to test an AI explanation rather than simply collect it.

### 3.2 Case and question generation

Teachers and students also use AI to draft cases, short-answer questions and multiple-choice items. This use can save time and can produce variations of a case that support practice. Recent work has examined AI-supported case and question generation in pharmacology and rational pharmacotherapy education (33–35). Student- or AI-generated case drafts also carry credibility risks, a concern echoed in problem-based learning contexts where students have reported perceived value but raised concerns about the credibility of generated information (36).

The risk is that the case may look plausible while omitting the variable that makes the medication decision safe or unsafe. A hypertension case without asthma history, renal function, pregnancy status or concurrent medicines may train students to answer too quickly. AI-generated cases should therefore be treated as drafts. Teachers still need to check the learning objective, pharmacological accuracy and safety boundary before using them in class.

### 3.3 Tutoring and feedback

AI can provide immediate hints, self-tests and explanatory feedback (37, 38). This is attractive in large classes where teacher feedback is limited. Reviews in health-professions education describe AI as a possible support for individualized learning and feedback (5).

Feedback, however, is only helpful when it improves the learner’s next attempt. If the tool supplies a complete answer too early, students may skip retrieval, comparison and correction (39). Formative assessment literature has long emphasized feedback that makes the gap between current and desired performance visible (40, 41). In pharmacology, AI feedback should therefore be designed around revision: ask for a source, ask for a mechanism chain, ask what patient variable changes the answer, and then revise.

### 3.4 Drug-information queries

Medication-related queries carry particular risk in pharmacology education. Students may ask for indications, contraindications, interactions, dose adjustment, adverse reactions or monitoring. Several studies show why this use requires caution. AI-generated medication answers can be incomplete, wrong, unsupported or inconsistent across prompts (13–15). Clinical-pharmacy evaluations also show uneven performance across drug-interaction detection, prescription review, adverse-drug-reaction recognition and complex pharmacy problem solving (16, 17, 42, 43). In pharmacy education, student-facing drug information activities have made learners more aware of reliability and over-reliance concerns (44, 45). Comparative studies also suggest that AI performance is weaker on applied or case-based pharmacy tasks than on simple recall tasks (46–48).

For pharmacology education, these findings point to a clear instructional requirement. Any AI-generated drug claim that affects safety or reasoning should be checked against a reliable source before it is used in a student answer. This requirement is not an optional evidence-based extension; it is part of learning how drug information should be handled.

### 3.5 AI literacy and academic integrity

AI literacy includes prompt design, disclosure, privacy protection, recognition of hallucination, awareness of automation bias and appropriate use of generated text. These are now discussed across health-professions education (19–21, 49). Pharmacy-education studies and reviews also show that students and faculty are already forming views about AI usefulness, reliability and limits (22–25). In pharmacology, however, AI literacy needs a sharper disciplinary focus.

A broad warning that AI may be wrong is not enough. Students need to know which claims demand checking: drug names, dose ranges, contraindications, interactions, special populations, monitoring and reference authenticity. They also need repeated practice in finding where a generated answer is wrong, incomplete or too general. That practice has to be built into coursework rather than left to individual judgment.

These five use cases do not carry the same level of pharmacological safety risk. Drug-information queries pose the risk because they may directly affect contraindications, interactions, dose adjustment or monitoring. Case-based reasoning and mechanism explanation also carry risk because they shape how students justify medication decisions. Tutoring support and AI literacy or academic integrity tasks usually carry lower immediate safety stakes, but they still influence students’ habits of verification.

### 3.6 Why these limitations arise

These limitations are consistent with how LLMs generate responses. Because they produce text from statistical patterns rather than routinely verifying claims against authoritative sources, fluent answers may still be inaccurate or unsupported (50). Medication-related evaluations have documented incomplete or incorrect answers, while reference-generation studies have identified fabricated citations and low retrieval precision (14, 51). Performance also varies with model version and prompting strategy (52, 53). In cardiovascular physiology questions based on core concepts, ChatGPT-4 answered 83.33% correctly compared with 60% for ChatGPT-3.5, while some correct answers were still accompanied by incomplete explanations (54). Structured prompting improved consistency in medical question answering (55). Under-specified clinical prompts may therefore yield generalized answers that miss safety-relevant exceptions. These findings support requiring students to verify drug claims, reconstruct mechanism chains and reassess generated answers against patient context before using them in pharmacology coursework.

Taken together, the reviewed studies show that medication-related answers may be inaccurate or incomplete, references may be unsupported or fabricated, performance may vary across models and prompts, and learners may value AI support while remaining uncertain about its reliability and appropriate use. These findings inform the course-level analysis that follows. The five problems in Section 4 describe how such limitations may affect formative pharmacology coursework.

## 4 Problems that need a course-level response

The uses described above create different classroom situations. A reminder that AI should be used responsibly will not by itself show whether a student checked a contraindication, understood a mechanism or used the patient history in a case. For formative pharmacology coursework, five problems require a process that produces visible learning evidence (Table 1).

### 4.1 Hidden AI use

When formative tasks are assessed only through final text, teachers cannot know whether the student used AI for brainstorming, explanation, answer generation or editing. A strict ban may be unrealistic, but unstructured permission also fails. A course needs a simple record of what the tool was asked to do and which parts of the response were kept.

### 4.2 Drug claims without source checking

A fluent answer may list contraindications or interactions with confidence while omitting the very exception that matters. This is especially dangerous for drug information because the claim may look like ordinary factual knowledge. Pharmacology coursework should separate generated claims from checked claims.

### 4.3 Mechanism as prose rather than reasoning

Students often copy a mechanism explanation as prose. The educational target is different. They should be able to connect targets, pharmacodynamic or pharmacokinetic changes, therapeutic effects, adverse reactions, contraindications and monitoring. This chain is what allows transfer from one case to another.

### 4.4 Patient context treated as decoration

In many case answers, age, asthma history, renal function, pregnancy status or concomitant medicines are mentioned but not used. AI can reinforce this problem by giving a general medication suggestion that sounds reasonable in isolation. Pharmacology courses should require students to show exactly how a patient variable changes the answer.

### 4.5 Final-answer assessment

If only the final answer is graded, the most important learning work remains invisible. Prompts, source checks, mechanism diagrams, correction notes and brief reflections are not extra paperwork when used carefully; they are evidence of how the answer was built.

## 5 A source-checking process framework for AI-permitted formative pharmacology coursework

On the basis of the problems above, we propose a five-step process framework for undergraduate formative pharmacology coursework in which AI use is allowed (Figure 1). The framework is deliberately simple. It avoids treating AI as a tutor that must be trusted or an offender that must be detected. Instead, it places generated text inside the ordinary discipline of pharmacology: claims must be sourced, mechanisms must be rebuilt and patient variables must be used.

### 5.1 Define the task and record AI use

Students first define the learning task and state what they used AI for. The task may be to clarify a mechanism, compare drug classes, draft a practice question, identify possible interactions or improve wording. The record should include the tool, the prompt and the part of the output that influenced the submitted work. The purpose is to make the learning process visible, not to punish permitted use.

### 5.2 Check drug claims with defined sources

Students then identify the drug claims that matter for safety or reasoning. These include mechanisms, indications, contraindications, dose adjustment, adverse reactions, interactions, special populations and monitoring. Each key claim should be checked against a designated source. For a beginner pharmacology course, source checking is most reproducible when the teacher provides a small source set rather than leaving students to search the open web without guidance.

A practical source hierarchy should be defined before the task and organized according to the type of claim being checked. For basic pharmacological mechanisms, the course textbook, lecture material or an assigned review is usually the most appropriate starting point because these sources align with the terminology, depth and learning objectives of the course. Official drug labels or regulatory product monographs should be used for drug-specific contraindications, warnings, dosing and routes of administration. Current clinical guidelines or local protocols are more appropriate for therapeutic choices within a disease context, while curated drug-information databases can support the evaluation of interactions, monitoring requirements and special populations. Primary studies and high-quality reviews become particularly important when a claim is disputed, newly emerging or insufficiently addressed by the designated course sources. Drug-information education has long emphasized that resource selection should be guided by the nature of the question and the type of information required (56).

This hierarchy is therefore claim-specific and pedagogically defined rather than a universal ranking of scientific evidence. Course materials are placed first for basic mechanisms because they provide a consistent foundation for novice learners, but they do not replace official product information for drug-specific safety statements, current guidelines for treatment decisions or primary evidence when a claim requires deeper appraisal.

Apparent disagreement between sources may arise because they address different parts of the same clinical decision. For example, when *β*-blockade is being considered for hypertension in a patient with asthma, a course source may explain why β1-selective blockers have less effect on airway β2 receptors than nonselective agents. Respiratory evidence may then qualify this statement by showing that the risk is reduced rather than absent, while hypertension guidelines determine whether a β-blocker should be selected as initial therapy when no compelling cardiovascular indication is present. These sources are complementary rather than directly contradictory because they address receptor selectivity, respiratory safety and therapeutic priority, respectively (see Section 6.1 for the worked example).

When a genuine discrepancy remains, students should document the conflicting statements, identify the source type and date, and judge which source is most authoritative, current and specific to the claim. Official or regulatory sources should take priority for product-specific safety information, whereas current guidelines should guide disease-specific treatment choices. Unresolved discrepancies should be brought to teacher discussion rather than resolved by selecting the source that best supports the original AI-generated answer. This approach provides students with a shared and reproducible standard for deciding whether a generated drug claim can be retained, revised or rejected.

### 5.3 Rebuild the pharmacological mechanism

The next step is to convert an explanation into a mechanism chain. For example, an explanation of *β*-blocker pharmacology should not be reduced to the simplistic claim that the drug class “lowers blood pressure.” Students should connect β1 blockade with cardiac and renal effects and β2 blockade with the risk of bronchoconstriction. Clinical reasoning literature shows that hidden reasoning processes have to be made explicit and practiced in context (57, 58). Pharmacological reasoning has the same need.

### 5.4 Test the answer against patient context

Students then return to the case. They ask whether age, pregnancy status, renal or hepatic function, allergy history, asthma, heart failure, infection severity or concomitant medicines alter the answer. This step prevents the common movement from “drug class” directly to “recommendation.” It also clarifies that patient variables are not background information but part of the pharmacological reasoning.

### 5.5 Revise with feedback and justification

Finally, students revise the answer. Revision should be based on source checking, mechanism reconstruction, peer discussion or teacher feedback. A short correction note is often enough: what was changed, why it was changed and which source or case variable justified the change. This makes the task assessable without requiring teachers to police every sentence.

## 6 Proposed teaching scenario and feasibility

A useful process must fit ordinary teaching conditions. Case-based learning exercises have already been used to promote AI readiness in pharmacology courses (59). The source-checking process can be introduced in one chapter before it is used across a course. Cardiovascular pharmacology is a suitable starting point because mechanism, contraindication and patient context are closely connected.

### 6.1 A proposed β-blocker activity in a patient with asthma

The following author-developed example illustrates the five-step process using a β-blocker question in a patient with asthma. The generated output, source-checking steps and revised answer were constructed for illustrative purposes and do not represent student data or findings from a teaching intervention.

A 45-year-old adult with established asthma uses an inhaled *β*2 agonist as rescue therapy and has recently been diagnosed with uncomplicated hypertension. The patient has no heart failure, previous myocardial infarction, angina, arrhythmia or other indication for β-blockade.

Before class, students could ask an AI tool: “Can a β-blocker be used to treat hypertension in a 45-year-old adult with asthma who uses an inhaled β2 agonist as rescue therapy? Explain the relevant receptor mechanisms and safety considerations.” The prompt and generated response would be reviewed with a teacher-provided source set.

An illustrative generated output might state: “Yes. A β1-selective blocker such as metoprolol can be used because it mainly blocks cardiac β1 receptors, lowers heart rate and cardiac output, and has little effect on bronchial β2 receptors. It is therefore generally safe for patients with asthma.” The answer is plausible but incomplete. It treats β1 selectivity as nearly absolute, does not acknowledge that selectivity may diminish as the dose increases, and does not consider whether a β-blocker is an appropriate initial treatment for uncomplicated hypertension when no separate cardiovascular indication is present.

Table 3 applies the five steps to this response. The pharmacological mechanism is checked first, followed by evidence on respiratory safety and the response to inhaled *β*2 agonists (60, 61). The 2024 Elevated Blood Pressure and Hypertension guideline is then used to determine whether the ability of a *β*-blocker to lower blood pressure makes it an appropriate initial choice in this case (62).

**Table 3:** Worked example of the five-step source-checking process for a *β*-blocker question in a patient with asthma.

Framework stage	Illustrative content	Educational function
Initial generated output	Yes. A β1-selective blocker such as metoprolol can be used because it mainly blocks cardiac *β*1 receptors, lowers heart rate and cardiac output, and has little effect on bronchial β2 receptors. It is therefore generally safe for patients with asthma.	The response is plausible but overstates β1 selectivity and does not examine whether a β-blocker is the preferred initial treatment for uncomplicated hypertension.
1. Define the task and record AI use	Task: evaluate whether a β-blocker can be used to treat uncomplicated hypertension in a patient with asthma who uses an inhaled β2 agonist as rescue therapy. Students record the AI tool, prompt and retained output.	Defines the question raised by the generated answer and documents how the output entered the coursework.
2. Check drug claims with defined sources	Mechanism: The assigned pharmacology source confirms that cardiac and renal β1 blockade reduces heart rate, contractility and renin release, thereby lowering cardiac output and renin–angiotensin system activity. Respiratory safety: Available evidence indicates that β1-selective blockers have a lower, but not absent, respiratory risk and may still reduce the bronchodilator response to inhaled β2-agonists (60, 61). Treatment choice: The 2024 hypertension guideline does not recommend β-blockers as preferred initial therapy for uncomplicated hypertension in the absence of a compelling cardiovascular indication (62).	Separates the questions of pharmacological effect, respiratory safety and therapeutic priority, each of which requires a different source type.
3. Rebuild the pharmacological mechanism	Cardiovascular pathway: cardiac and renal β1 blockade → reduced heart rate, contractility and renin release → lower cardiac output and renin-angiotensin system activity → lower blood pressure. Airway pathway: inhaled β2 agonist → bronchial β2-receptor activation → smooth-muscle relaxation. Nonselective or residual β2 blockade → reduced β2 signaling → bronchoconstriction and a weaker bronchodilator response.	Shows why a drug can lower blood pressure while still creating an airway safety concern.
4. Test the answer against patient context	The patient has asthma requiring intermittent β2 agonist rescue treatment and has no compelling cardiovascular indication for β-blockade. Although a β1-selective agent may sometimes be used in asthma, this does not make it the preferred initial treatment for uncomplicated hypertension. Guideline-supported first-line options include an ACE inhibitor or ARB, a dihydropyridine calcium-channel blocker, or a thiazide or thiazide-like diuretic (62).	Uses the asthma history and the absence of a separate β-blocker indication to refine the treatment decision.
5. Revise with feedback and justification	Revised answer: A β-blocker can lower blood pressure, but it would not usually be selected as the initial treatment in this case. A nonselective β-blocker should generally be avoided because airway β2 blockade may provoke bronchoconstriction and reduce the response to the patient’s inhaled β2 agonist. A β1-selective blocker has a lower respiratory risk, but its selectivity is incomplete and dose dependent. Because the patient has uncomplicated hypertension and no separate cardiovascular indication for β-blockade, an ACE inhibitor or ARB, a dihydropyridine calcium-channel blocker, or a thiazide or thiazide-like diuretic would generally be preferred as initial therapy. If a compelling cardiovascular indication for β-blockade later arises, a β1-selective agent may be considered cautiously with respiratory monitoring.	Links the revised recommendation to the checked mechanism, respiratory evidence, hypertension guideline and patient context.

Mechanism reconstruction shows why the answer cannot be reduced to receptor selectivity alone. Cardiac and renal β1 blockade lowers heart rate, contractility and renin release. In the airway, β2-receptor activation by the patient’s rescue inhaler promotes bronchodilation; nonselective β-blockade can oppose this effect, while β1-selective agents have a lower but not absent respiratory risk.

After the case details and treatment guideline are considered, the conclusion changes. A β-blocker can lower blood pressure, and a *β*1-selective agent may sometimes be used in a patient with asthma, but neither point establishes that it should be selected for uncomplicated hypertension. An angiotensin-converting enzyme inhibitor or angiotensin receptor blocker, a dihydropyridine calcium-channel blocker, or a thiazide or thiazide-like diuretic would generally be preferred as initial therapy. If a compelling cardiovascular indication for β-blockade later arises, a β1-selective agent may be considered cautiously with respiratory monitoring.

### 6.2 Practical formats for large classes

The full process does not have to be used in every learning task. In a large class, teachers can ask students to check only three key AI-generated claims, submit one mechanism chain and write a 100-word revision note. Peer checking can be used for the first pass, while the teacher samples selected submissions or focuses on claims most likely to affect safety. A 20- to 30-min classroom activity is usually enough for the *β*-blocker example if the prompt and initial answer are prepared before class. Table 4 summarizes feasible implementation formats that vary in task length, suitable use and teacher workload control.

**Table 4:** Feasible implementation formats.

Format	Suitable use	Teacher workload control
Short in-class activity	One case in a chapter; 20–30 min after pre-class AI prompting.	Students check three key claims; teacher discusses common errors.
Chapter formative task	One-page submission after cardiovascular, antimicrobial or CNS pharmacology.	Use a fixed template and peer checking before the teacher reviews a sample of submissions.
Extended coursework task	Multiple cases across chapters or a small portfolio of source-checking tasks.	Use a rubric, predefined source sets, peer review and inter-rater calibration.

### 6.3 Transparency, honesty and teacher workload

Transparency is more likely when disclosure is treated as part of the coursework task rather than as a confession. Students should know in advance which uses are permitted, which are prohibited and how disclosure will be graded. Courses can predefine acceptable uses, such as clarifying mechanisms, drafting initial responses or polishing language, and prohibited uses, such as submitting full AI output as original work, using AI to complete all case reasoning or entering identifiable patient information into public AI tools.

The framework is intended for AI-permitted formative coursework. It is not an AI-detection system and should not be used as the main safeguard for high-stakes closed-book assessment. Its safeguard is pedagogical: if a student uses AI but provides no task record, source check, mechanism chain or revision note, even a fluent final answer cannot receive a high score on the process dimensions. In this way, the design lowers the reward for hiding AI use by making process evidence part of the grade. For teachers, workload can be limited by using templates, source sets, peer review and focused rubrics. The aim is not to detect every AI sentence. The aim is to ask students to defend important pharmacological claims.

## 7 Assessment approach

Assessment should follow the same five stages as the framework rather than treating the revised answer as the only product. The proposed analytic rubric therefore evaluates: (1) task definition and AI-use record, (2) source checking, (3) mechanism reconstruction, (4) patient-context appraisal and (5) revision with justification. Each dimension should be scored separately so that a polished answer cannot compensate for missing evidence of checking or reasoning.

Table 5 provides descriptors for low, developing and satisfactory performance. At the satisfactory level, the AI-use record identifies the tool, prompt, purpose and retained material; source checking matches important claims to appropriate sources and explains discrepancies; the mechanism chain is pharmacologically accurate; patient variables are used to alter or qualify the judgment; and the revision note identifies what changed, why it changed and what evidence justified the change. Supplementary Table S1 further provides author-developed examples at low, developing and satisfactory levels for the β-blocker/asthma task (they were constructed to make the rubric concrete and are not responses collected from students). The reliability and validity of these descriptors should be examined in future classroom studies.

**Table 5:** Proposed analytic rubric aligned with the five-step framework.

Dimension	Low	Developing	Satisfactory
1. Define the task and record AI use	Tool, prompt or retained output is missing; purpose of use is unclear.	Required elements are present but incomplete or do not show how output influenced the work.	Tool, prompt, purpose, retained output and influence on the submission are clearly documented.
2. Check drug claims with defined sources	Important drug claims are unchecked, sources are inappropriate or fabricated, or discrepancies are ignored.	Most key claims are checked, but source choice or conflict handling is incomplete.	Safety- and reasoning-relevant claims are matched to appropriate sources; discrepancies and source choice are explained.
3. Rebuild the pharmacological mechanism	Mechanism is copied as prose or contains major pharmacological errors.	A causal chain is attempted but contains omissions or weak links.	An accurate chain connects the relevant target with downstream pharmacological effects, therapeutic benefit or risk, and monitoring where relevant.
4. Test the answer against patient context	Patient variables are absent or merely listed.	At least one variable is linked to the answer, but its effect on judgment is incomplete.	Relevant variables are used explicitly to change or qualify the medication judgment or to determine appropriate monitoring.
5. Revise with feedback and justification	No meaningful revision or rationale is provided.	The answer is revised, but the reason or supporting evidence is partly unclear.	The revision identifies what changed, why it changed and which source or case variable justified the change.

Scoring reliability can be supported by calibrating two or three anchor examples before marking, using a fixed source-checking template and reviewing a sample of scores across instructors or peer reviewers. In large classes, peers may check whether required fields are present, while teachers focus on contested drug claims, the mechanism chain and the final justification. The assessment weight should remain proportional to the task: a short in-class activity may be completion-based, whereas a chapter-level formative task can use the full analytic rubric.

## 8 Discussion

This review argues that AI should be handled in pharmacology education as a source of provisional material. The educational focus is how students evaluate and revise generated material. In pharmacology coursework, this response can be organized around claim checking, mechanism reconstruction and patient-context appraisal.

The incremental contribution of the proposed process becomes clearer when it is compared with existing work. Current pharmacy and health-professions AI literature has described student perceptions, faculty attitudes, AI performance, AI-assisted case generation and broad guidance on academic integrity. These contributions are useful, but they do not always specify what evidence a student should submit after using AI in a formative pharmacology task. Conventional clinical pharmacology reasoning instruction, by contrast, teaches students to connect drug mechanisms with patient variables, but it was not designed for a setting in which a generated answer may contain unsupported claims, fabricated references or hidden omissions. The present process adds an AI-specific layer to pharmacology reasoning: task recording, generated-claim source checking, explicit source hierarchy, mechanism reconstruction from generated prose, and a revision note that shows how the answer changed.

Three design choices give the process its pharmacology-specific value. First, source checking is treated as a graded drug-safety task rather than a general fact-checking instruction. Students are asked to match the type of claim with an appropriate source, because a receptor mechanism, a boxed warning, a dose-adjustment statement and a guideline-based therapeutic choice do not carry the same evidentiary requirements. Second, mechanism reconstruction is required because students may be able to repeat a mechanism without explaining how a target leads to therapeutic benefit, risk, contraindication or monitoring. The process therefore asks students to turn generated prose into a mechanism chain. Third, patient-context testing is mandatory. A medication suggestion that is broadly correct may still be unsafe or incomplete when renal function, pregnancy, asthma, allergy, concomitant medicines or monitoring needs are considered. These elements are often less explicit in generic AI-literacy frameworks, and they explain why the process is more than an AI-use checklist.

Source definition is central to reproducibility. Without a shared source rule, one student might check a contraindication against a textbook, another against an AI answer with a citation, and a third against an outdated webpage. The proposed hierarchy is not meant to settle every clinical controversy. It gives undergraduate courses a common starting point: course sources for mechanisms, official labels for product-specific safety information, guidelines or local protocols for context-dependent therapeutic choices, curated drug-information databases for interaction and monitoring checks, and higher-level reviews or primary studies when a claim is contested. This makes the process easier to teach, compare and grade.

The approach also differs from AI-detection strategies. Detection asks who wrote the text. The more useful educational question is whether the student can justify the drug claim and mechanism in front of a teacher or peer. This distinction matters because AI-generated writing may become increasingly difficult to recognize, while the need to defend pharmacological reasoning will remain.

Several limitations should be considered. The proposed process was developed from literature synthesis and educational reasoning rather than classroom testing; its effects on pharmacological reasoning, student acceptability, teacher workload, scoring reliability and long-term learning are therefore unknown. The worked output, rubric anchors and supplementary submission profiles are author-developed examples rather than student data. As a narrative review, the search and selection process was guided by thematic relevance to pharmacology coursework and did not include a formal risk-of-bias appraisal, which may introduce selection bias. The process is intended for AI-permitted formative coursework in undergraduate pharmacology and should not replace safeguards in high-stakes summative examinations, clinical placement assessment, licensing decisions or direct patient-care training. Future studies should pilot the process, examine inter-rater agreement and compare ordinary AI use, no-AI tasks and source-checking tasks across chapters and class sizes.

## 9 Conclusion

AI has become part of the learning environment for undergraduate pharmacology. The main educational challenge is not the existence of generated answers, but the possibility that students may accept them without checking evidence, mechanism or patient context. This narrative review proposes a source-checking process that asks students to record AI use, verify drug claims against defined sources, rebuild the pharmacological mechanism, test the answer against patient context and revise with feedback and justification. Used selectively, the process may help teachers preserve the core goals of pharmacology education while giving students a practical way to use AI with discipline-specific caution. Its feasibility and effects should be tested in classroom practice.
