# Supplementary material for: Guiding student use of generative AI in undergraduate pharmacology: a narrative review and proposed source-checking process framework
Source: Front Med (Lausanne). 2026 Jul 28;13:1910543. doi: 10.3389/fmed.2026.1910543 (PMC13458756; doi:10.3389/fmed.2026.1910543)
Supplement: Supplementary file 1 [file Table_1.docx]

Supplementary Table S1. Author-developed scoring anchors for the β-blocker/asthma worked example presented in Table 3

| Performance level | Illustrative submission | Rationale |
| --- | --- | --- |
| Low | **AI-use record:** Not provided.  **Source checking:** No key drug claims are checked.  **Mechanism:** The response states only that β-blockers lower blood pressure, without explaining the β1-mediated cardiovascular pathway or the airway consequences of β2 blockade.  **Patient context:** Asthma is mentioned but does not affect the medication judgment.  **Revision:** No meaningful revision is made. The response repeats that metoprolol can be used because it is β1 selective and therefore safe in asthma. | Core process evidence is missing. The response repeats the generated conclusion, treats β1 selectivity as absolute, and does not examine whether a β-blocker is the preferred initial treatment for uncomplicated hypertension. |
| Developing | **AI-use record:** The AI tool and prompt are recorded, but the retained output, purpose of use, or influence on the submission is incompletely documented.  **Source checking:** The mechanism and respiratory-safety claims are checked, but guideline-based therapeutic priority is not verified, or the choice of source is not fully justified.  **Mechanism:** β2 blockade is linked to bronchoconstriction, but the cardiovascular pathway is incomplete; for example, the effect of β1 blockade on renin release is omitted.  **Patient context:** Asthma is linked to respiratory caution, but the absence of a compelling cardiovascular indication for β-blockade is not used to change the initial treatment decision.  **Revision:** The answer is changed to state that a low dose of metoprolol could be used with caution, but the reason for retaining a β-blocker and the supporting evidence remain incomplete. | All five stages are attempted, but source selection, mechanism reconstruction, and patient-context appraisal remain incomplete. The revised answer adds respiratory caution without resolving whether β-blockade is indicated for this patient. |
| Satisfactory | **AI-use record:** The AI tool, prompt, purpose, retained output, and how the output influenced the submission are clearly documented.  **Source checking:** The cardiovascular mechanism, respiratory safety, and therapeutic choice are checked against appropriate source types. Differences between the generated answer and the checked sources are documented, and the source selected for each claim is justified.  **Mechanism:** Separate cardiovascular and airway pathways are accurately reconstructed. The response links cardiac and renal β1 blockade to reduced heart rate, contractility, and renin release, and links β2 blockade to bronchoconstriction and a reduced bronchodilator response. Appropriate respiratory monitoring is identified if β-blockade becomes necessary.  **Patient context:** Asthma, use of an inhaled β2 agonist as rescue therapy, and the absence of a compelling cardiovascular indication for β-blockade are explicitly used to change the treatment decision.  **Revision:** The initial recommendation to use metoprolol is replaced with a guideline-supported first-line non-β-blocker class because the patient has asthma and no compelling cardiovascular indication for β-blockade. The response also explains that a β1-selective blocker could be considered cautiously if a separate cardiovascular indication later arises. | The submission integrates all five stages, corrects the overgeneralized generated answer, and links the final recommendation to the checked mechanism, respiratory evidence, hypertension guideline, and patient context. |

*Note: These author-developed examples illustrate how the analytic rubric in Table 5 may be applied to the worked example in Table 3. Each rubric dimension should be scored separately. The labels low, developing, and satisfactory describe illustrative submission profiles and are not based on actual student responses or empirical outcomes.*
